# Radiation exposure in cardiac computed tomography imaging in Mie prefecture in 2021

**DOI:** 10.1007/s11604-022-01380-0

**Published:** 2023-01-06

**Authors:** Suguru Araki, Kakuya Kitagawa, Takanori Kokawa, Masafumi Takafuji, Satoshi Nakamura, Naoki Nagasawa, Hajime Sakuma

**Affiliations:** 1 Department of Radiology, Mie University Hospital, 2-174 Edobashi, Tsu, Mie 514-8507 Japan; 2 Department of Advanced Diagnostic Imaging, Mie University Graduate School of Medicine, 2-174 Edobashi, Tsu, Mie 514-8507 Japan; 3 Faculty of Health Science, Suzuka University of Medical Science, 1001-1, Kishioka, Suzuka, Mie 510-0293 Japan

**Keywords:** Cardiac computed tomography, Radiation dose exposure, Dose-length product, Electrocardiogram-triggered prospective scanning, Tube potential

## Abstract

**Purpose:**

Several effective radiation dose reduction methods have been developed for coronary computed tomography angiography (CTA); however, their use in daily clinical practice remains unknown. We aimed to investigate radiation exposure and the utilization of dose-saving strategies for coronary CTA in hospitals in Mie Prefecture, Japan.

**Materials and methods:**

Image acquisition details and dose reports of 30 consecutive cardiac CT examinations performed in 2021 were obtained from 18 hospitals. The inclusion criteria were patients aged 20–80 years who weighed 50–70 kg and underwent coronary CTA using ≥ 64-row multidetector CT. The doses for the overall cardiac CT examination and coronary CTA were analyzed using the dose-length product (DLP) and CT dose index (CTDIvol), respectively. Multivariate analysis was performed to determine independent predictors that affect the radiation dose in coronary CTA.

**Results:**

The median DLP of cardiac CT was 774 (interquartile range [IQR]: 538–1119) mGy*cm, and the median CTDIvol of coronary CTA was 33 (IQR: 25–48) mGy. The 75th percentile values of DLP for cardiac CT and that of CTDIvol for coronary CTA were slightly lower than the values recorded in the Japan Diagnostic Reference Level (DRLs) 2020 report (1285 mGy*cm and 66.4 mGy, respectively) but were substantially higher than those reported in a previous large international dose survey (402 mGy*cm and 24 mGy, respectively). Iterative reconstruction was performed during all examinations. Only six hospitals (33%) used a low tube potential (≤ 100 kVp), and nine hospitals (50%) used electrocardiogram-triggered prospective scanning. Multivariate analysis revealed low heart rate, low tube potential, and use of electrocardiogram-triggered prospective scanning as independent predictors of CTDIvol ≤ 24 mGy (*p* < 0.001, respectively).

**Conclusion:**

As of 2021, low tube potential and prospective scanning are underutilized, whereas iterative reconstruction is used in every coronary CTA in Mie Prefecture. Further efforts to optimize the radiation exposure from cardiac CT scans are necessary.

**Supplementary Information:**

The online version contains supplementary material available at 10.1007/s11604-022-01380-0.

## Introduction

Cardiac computed tomography (CT) is widely used as a non-invasive imaging modality in clinical practice [1, 2]. Coronary CT angiography (CTA) has high diagnostic accuracy in the identification of coronary artery stenosis and can help rule out coronary artery disease (CAD) with a high negative predictive value [3]. Furthermore, coronary CTA provides significant prognostic information and may allow for a significant reduction in cardiac deaths or non-fatal myocardial infarctions [4, 5]. According to a survey by the Japanese Cardiovascular Society, approximately 500,000 coronary CTA examinations are performed annually in Japan [6]. This number is expected to increase in future as the 2022 Japanese Circulation Society Guidelines have identified coronary CTA as the first-line test for stable CAD [7].

Radiographic examinations, including cardiac CT, should be optimized to ensure that the dose is “as low as reasonably achievable (ALARA)” [8]. In 2007, cardiac CT data from 64 global hospitals were collected in the international dose survey PROTECTION I, which revealed a high median dose-length product (DLP) of 885 mGy*cm and large inter-site variability (interquartile range [IQR]: 560–1239 mGy*cm) [9]. However, due to the widespread use of dose-saving technologies, the radiation dose for cardiac CT is now a quarter of that recorded in 2007 with a median DLP of 195 (IQR: 110–338 mGy*cm) observed in PROTECTION VI [10]. Conversely, in the Japan Diagnostic Reference Level (DRLs) 2020 report, a radiation dose survey reported that the DRL of cardiac CT was 1300 mGy*cm [11]. This is the same level as that reported in PROTECTION I in 2007 but 3.25 times higher than the DRL of 400 mGy*cm estimated in PROTECTION VI. It is unclear why the radiation doses for cardiac CT examinations are higher in Japan than in other countries where drastic dose reductions have been achieved in the last decade.

Therefore, we performed a dose survey to investigate the radiation dose of cardiac CT in real-world clinical practice in Japan.

## Materials and methods

### Study protocol

This study was approved by the Clinical Research Ethics Review Committee of Mie University Hospital (approval No. H2021-206). The requirement for written informed consent was waived because this study used existing clinical data.

We surveyed institutions in Mie Prefecture that have multidetector row computed tomography (MDCT) scanners with 64 or more rows and identified 44 institutions in December 2020. These 44 institutions were then surveyed, and the number of cardiac CT examinations performed in 2021 was recorded in the primary survey. Responses were received from 34 institutions; of them, 24 institutions had performed cardiac CT. We requested the 24 institutions to cooperate in the secondary survey; of them, 21 institutions (all hospitals) agreed.

In the secondary survey, local collaborators collected the image acquisition details and dose reports of 30 consecutive patients who underwent cardiac CT as part of routine clinical practice from January to December 2021. The inclusion criteria were as follows: patients aged 20–80 years who weighed 50–70 kg and underwent coronary CTA using ≥ 64-row MDCT. The inclusion criteria of age and weight were set as same as those of Japan DRLs 2020 to facilitate comparison with the study. The exclusion criteria were as follows: patients who underwent any special scanning protocol for other clinical studies, incomplete CT studies due to adverse events during scanning, and incomplete datasets.

**Fig. 1:**
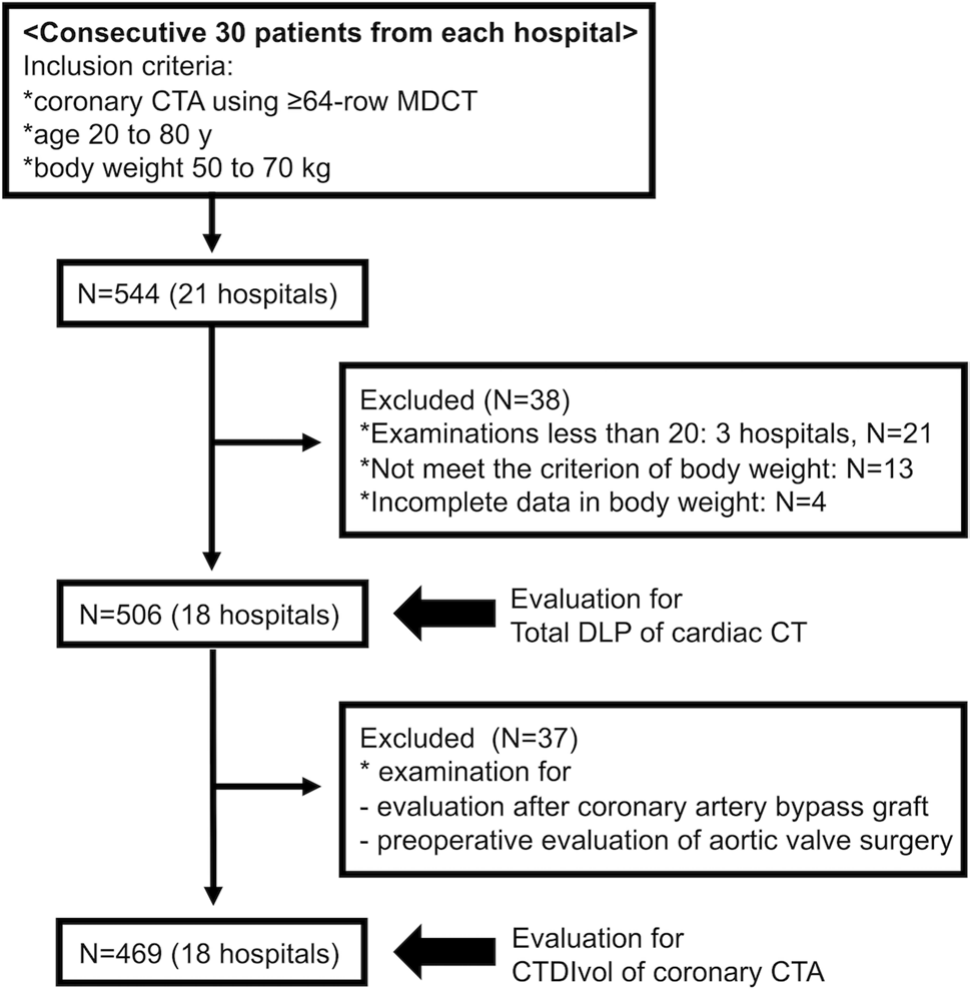
Flowchart depicting patient selection. *CTA* computed tomography angiography, *MDCT* multidetector row computed tomography, *DLP* dose-length product, *CTDIvol* computed tomography dose index volume

We obtained data from 544 patients from 21 hospitals. Three hospitals had fewer than 20 patients who underwent the imaging examinations; 21 patients from these hospitals were excluded from the following analysis according to the exclusion criteria of the Japan DRLs 2020. Thirteen patients who did not meet the criteria for body weight and four patients whose body weight was unknown were also excluded from the analysis (Fig. 1).

All observational data were analyzed in the core laboratory. The following patient characteristics were collected: sex, height, body weight, indication for cardiac CT (coronary artery evaluation or other indications), heart rate, heart rhythm (sinus rhythm or others), and the use of nitroglycerin and β-blocker formulations. The following imaging parameters were recorded: CT system (number of detector rows), tube potential, utilization of iterative image reconstruction (IR), utilization of automatic exposure control (AEC), utilization of electrocardiogram (ECG)-controlled dose modulation (DM), and scanning technique (ECG-gated retrospective scanning, CTA/cardiac function analysis [CFA], or ECG-triggered prospective scanning [axial or high-pitch helical]). CTA/CFA is an ECG-gated scan mode of Canon’s 320-row MDCT, which can capture the entire R-R interval without table movement. CTA/CFA, when performed in a single beat, behaves similarly to a ECG-triggered prospective axial scanning and requires a relatively low dose. However, CTA/CFA with two or three beats, which allows segmental reconstruction, behaves more like a ECG-gated retrospective scanning and involves 2–3 times the dose of a single-beat CTA/CFA.

### Radiation dose estimation

Parameters relevant to the radiation dose were obtained from the dose report and assessed using the volume CT dose index (CTDIvol) and DLP. The CTDIvol is calculated as the integral of the radiation dose profile of a single rotation scan. The DLP is calculated by multiplying the CTDIvol by the respective scan length and represents the radiation dose received during the entire CT scan. In this study, the total DLP of cardiac CT was all-inclusive (positioning, unenhanced scan, and non-cardiac scan) and represented the entire CT examination, while the CTDIvol of coronary CTA represented only the coronary CTA.

### Statistical analysis

Continuous data are expressed as median with IQR or count with percentage. To detect independent predictors associated with radiation dose in coronary CTA, we compared each parameter between the groups using the Wilcoxon–Mann–Whitney U test. Only variables with *P* < 0.10 on univariate analysis were included in the multivariate analysis to avoid overfitting the multivariable model. Differences in the CTDIvol per variable were assessed using the Wilcoxon–Mann–Whitney U test. In all tests, statistical significance was set at *P* < 0.05.

## Results

### Patient and study site characteristics

In total, 506 patients were recruited from 18 hospitals. Men accounted for 61% (*n* = 307) of patients, and the overall median age was 70 years (IQR: 62–75 years), the median height was 162 cm (IQR: 155.7–167.7 cm), the median weight was 61 kg (IQR: 55–65 kg), and 465 (92%) of the patients were examined for sinus rhythm (Table 1).

**Table 1:** Patient and study site characteristics

	*N* = 506
Age (year)	70 (62–75)
Sex, men, n (%)	307 (61%)
Height (cm)	162 (155.7–167.7)
Weight (kg)	61 (55–65)
Heart rate (beats/min)	60 (55–66)
Heart rhythm, *n* (%)	
Sinus rhythm	465 (92%)
Others	41 (8%)

Data are presented as the median (interquartile range) or number of patients (%)

*CTA* computed tomography angiography

The total number of beds was greater than 400 in nine hospitals (50%), 200–400 in seven hospitals (39%), and less than 200 in two hospitals (11%). The overall median duration of experience with cardiac CT studies was 12.5 years (IQR: 11–15 years), and the median number of cardiac CT studies was 16 per month (IQR: 10–25). Characteristics of each hospital are listed in Supplementary Table 1.

### Imaging characteristics and strategies for reduction of radiation dose

All studies were performed using CT scanners with 64 or more detector rows; 39% and 23% of scans were performed using area detector and dual-source CT scanners, respectively. Regarding the CT scanner, 55%, 29%, 11%, and 5% of the scans were performed using scanners from Canon (Otawara, Japan), Siemens (Forchheim, Germany), Philips (Best, The Netherlands), and GE (Milwaukee, WI, USA). The characteristics of the CT scanners used in this dose survey are listed in Supplementary Table 2. IR and AEC were used in all patients, whereas DM was used in 63% of the scans. ECG-gated retrospective scanning and ECG-triggered prospective scanning were utilized in 47% and 46% of the scans, respectively. Low tube potential scanning was used in 31% of the patients (Table 2).

**Table 2:** Scan characteristics

	*N* = 506
CT characteristics, *n* (%)	
Area (16 cm or 8 cm) -detector CT	196 (39%)
Dual-source CT	119 (23%)
Standard CT (single source, 4 cm -detector)	191 (38%)
CT manufacturer, *n* (%)	
Canon	276 (55%)
Siemens	149 (29%)
Philips	55 (11%)
GE	26 (5%)
Iterative image reconstruction (IR), *n* (%)	506 (100%)
Automatic exposure control (AEC), *n* (%)	506 (100%)
ECG-controlled dose modulation (DM), *n* (%)	320 (63%)
Scan technique, *n* (%)	
ECG-gated retrospective scanning	226 (47%)
CTA/CFA	37 (7%)
ECG-triggered prospective scanning	233 (46%)
Tube potential, *n* (%)	
135 kVp	2 (0.4%)
120 kVp	346 (68%)
100, 90 kVp	100 (20%)
80, 70 kVp	58 (11%)

Data are presented as number of patients (%)

*ECG* electrocardiogram, *CT* computed tomography, *CTA/CFA* continuous computed tomography angiography/cardiac function analysis

### Total DLP from cardiac CT and variation between hospitals

**Fig. 2:**
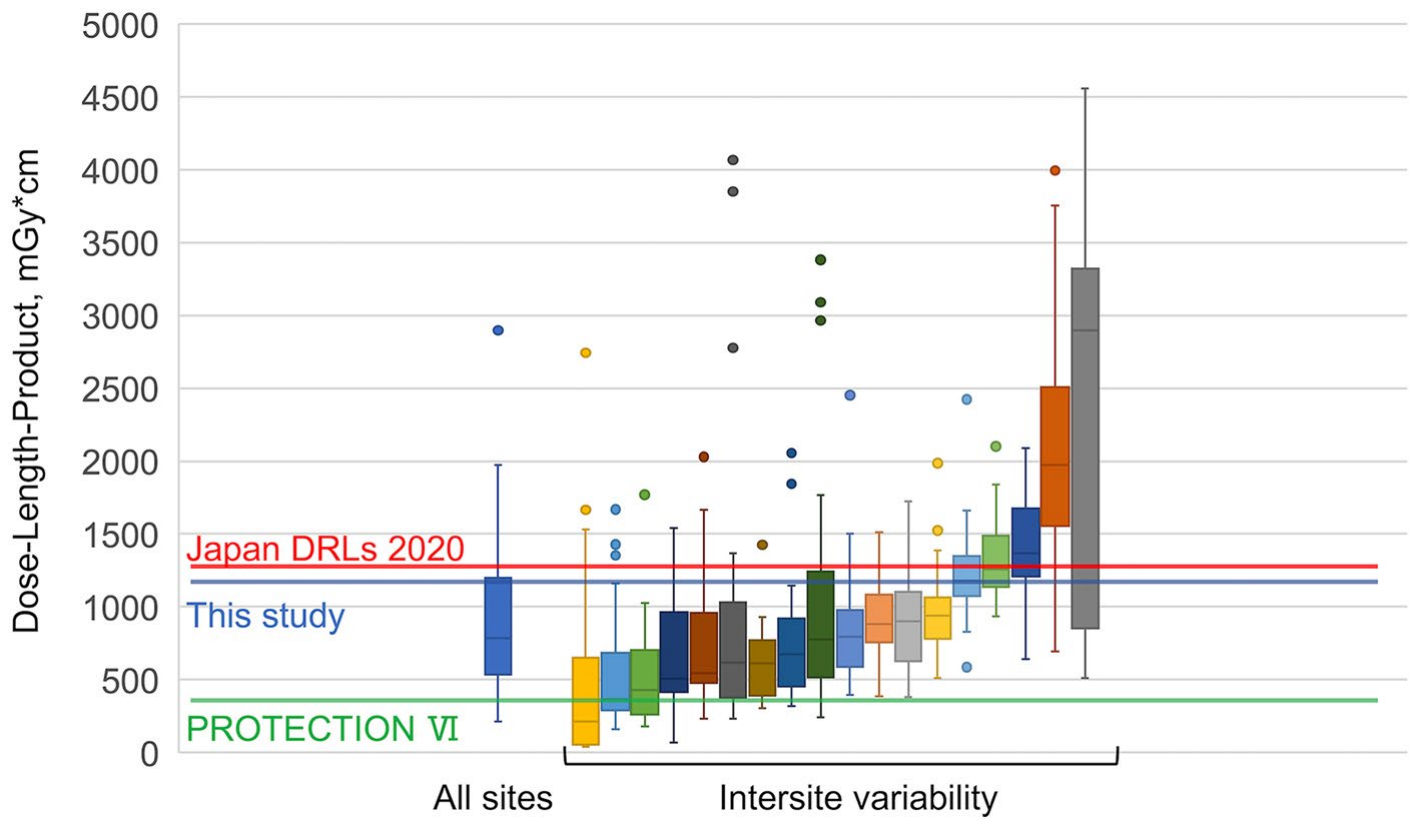
Total dose-length product of cardiac computed tomography at each site. DLP from cardiac CT and variation between study sites. Left: Box plot of the median DLP at all sites in Mie Prefecture. Right: Box plots of each site in Mie Prefecture. Box plots show the median dose-length product (± interquartile range). Red, blue, and green lines show the DRL of Japan DRLs 2020, this study, and PROTECTION VI, respectively. *DLP* dose-length product, *CT* computed tomography, *DRL* diagnostic reference level

Figure 2 illustrates the total DLP for cardiac CT scans at each hospital. The red line represents the DRL of the Japan DRLs 2020 (1300 mGy*cm), the green line represents the DRL of the PROTECTION VI study (400 mGy*cm), and the blue line represents the DRL in the present study (1119 mGy*cm). The DRL estimated from this study was slightly lower than that of the Japan DRLs 2020 but higher than that of PROTECTION VI. The total DLP included radiation dose not only from coronary CTA but also from positioning scans, non-contrast CT for calcium scoring and additional imaging of the chest, abdomen and pelvis, etc. We found that additional imaging was performed in about 30% of the all scans collected. Actually, five of the 18 hospitals performed extensive imaging of the chest, abdomen, and pelvis as part of a cardiac CT examination in > 50% of the collected cases. Therefore, it is possible that the total DLP does not reflect the actual dose in the coronary CTA examination portion.

### CTDIvol of coronary CTA and variation between study sites

**Fig. 3:**
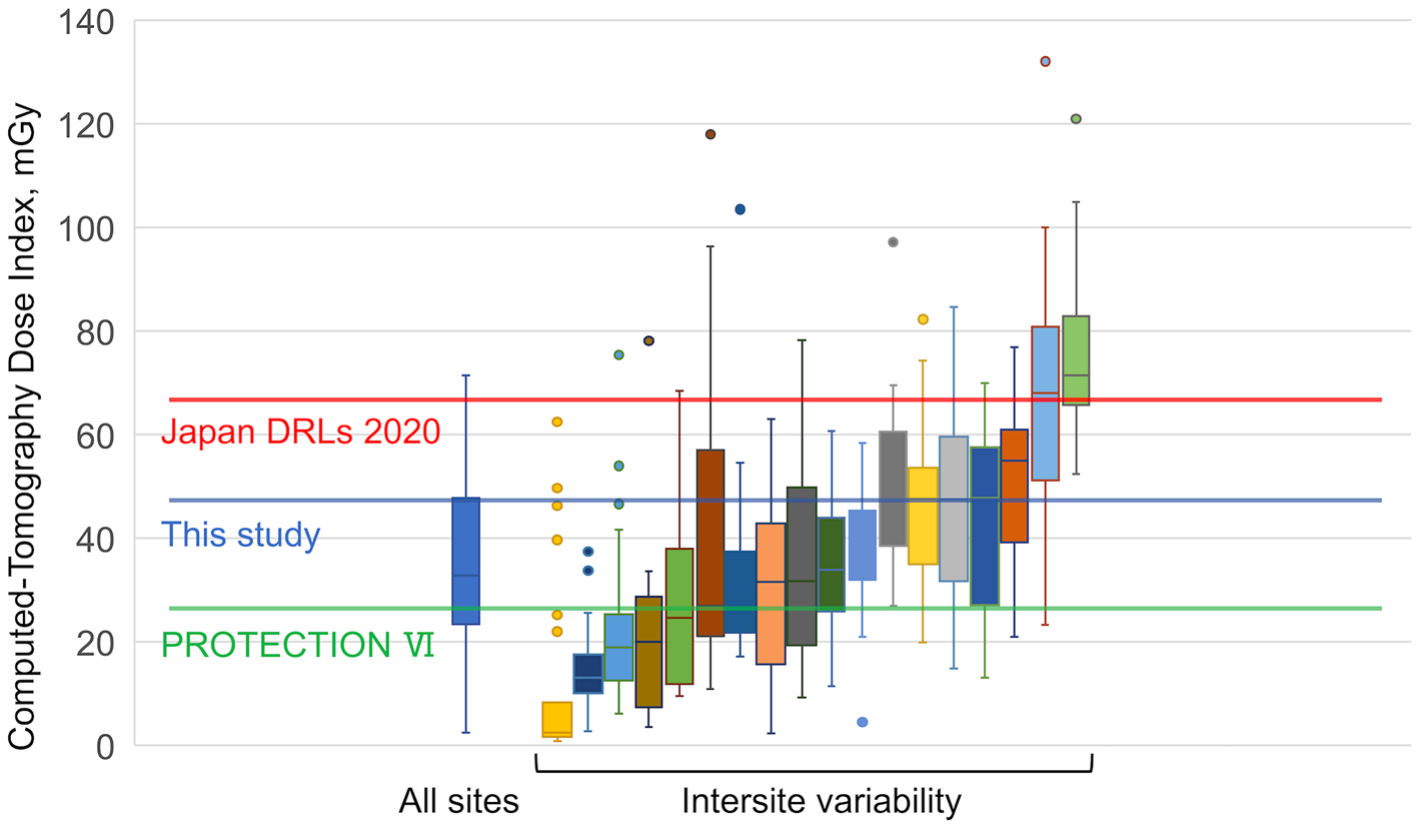
Computed tomography dose index of coronary computed tomography angiography at each site. CTDIvol from coronary CTA and variation between study sites. Left: Box plot of the median CTDIvol for all sites in Mie Prefecture. Right: Box plots of each site in Mie Prefecture. Boxplots show the median CTDIvol (± interquartile range). Red, blue, and green lines show the DRL of Japan DRLs 2020, this study, and PROTECTION VI, respectively. *CTDIvol* computed tomography dose index volume, *CTA* computed tomography angiography, *DRL* diagnostic reference level

To determine the radiation dose of coronary CTA for coronary artery evaluation, we evaluated the CTDIvol of coronary CTA (CTDIvol_CCTA_) after excluding 37 patients who underwent scanning for the evaluation of coronary artery bypass graft or preoperative evaluation for aortic valve surgery (Fig. 1). Figure 3 illustrates the CTDIvol_CCTA_ of each hospital. The red line represents the DRL of the Japan DRLs 2020 (66 mGy), the green line represents the DRL of the PROTECTION VI study (24 mGy), and the blue line represents the DRL of the present study (48 mGy). Similar to the total DLP from cardiac CT, the DRL of CTDIvol_CCTA_ estimated in this study was slightly lower than that of the Japan DRLs 2020 but higher than that of PROTECTION VI.

### Characteristics of scan technique, heart rate, and tube potential of each study site

**Fig. 4:**
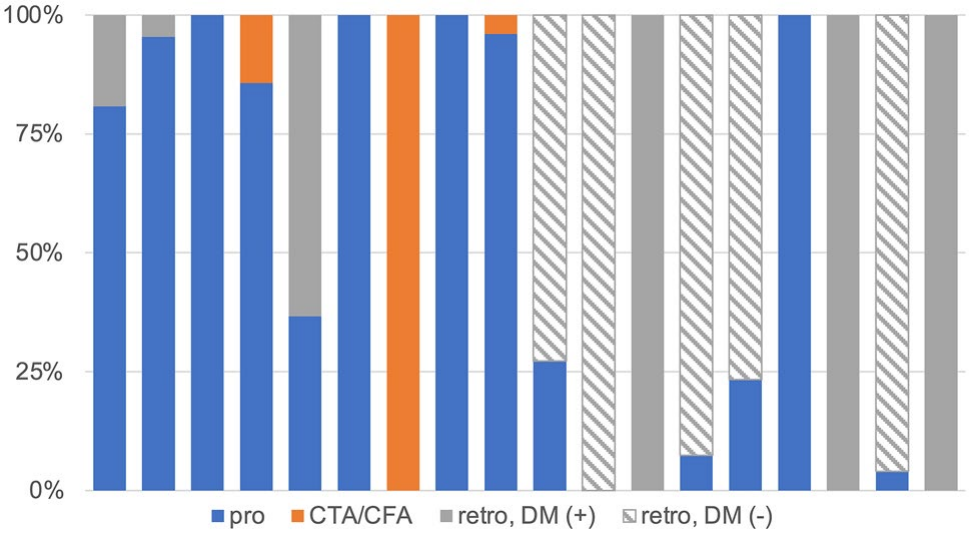
Scan techniques and electrocardiogram-controlled dose modulation at each site pro, electrocardiogram-triggered prospective scanning; retro, electrocardiogram-gated retrospective scanning; DM, electrocardiogram-controlled dose modulation

**Fig. 5:**
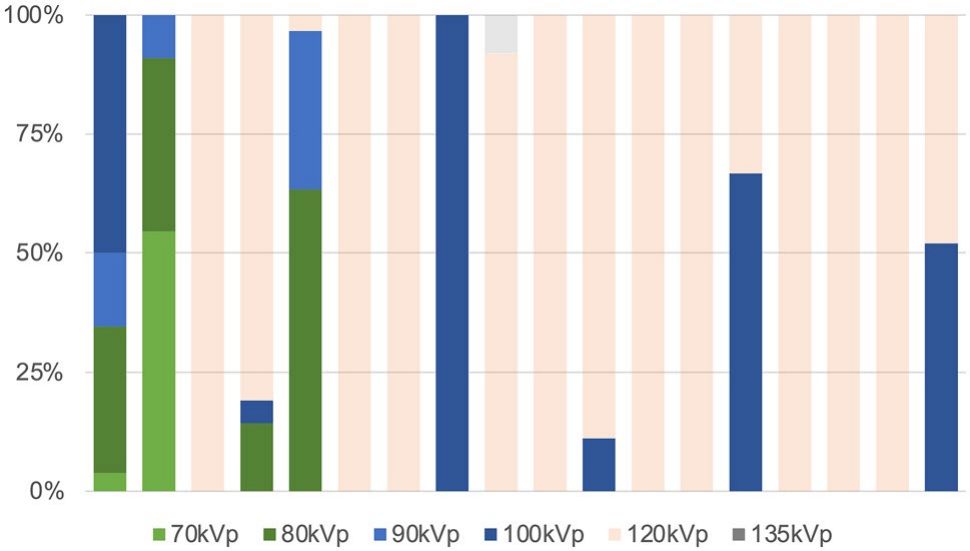
Tube potential at each site

**Fig. 6:**
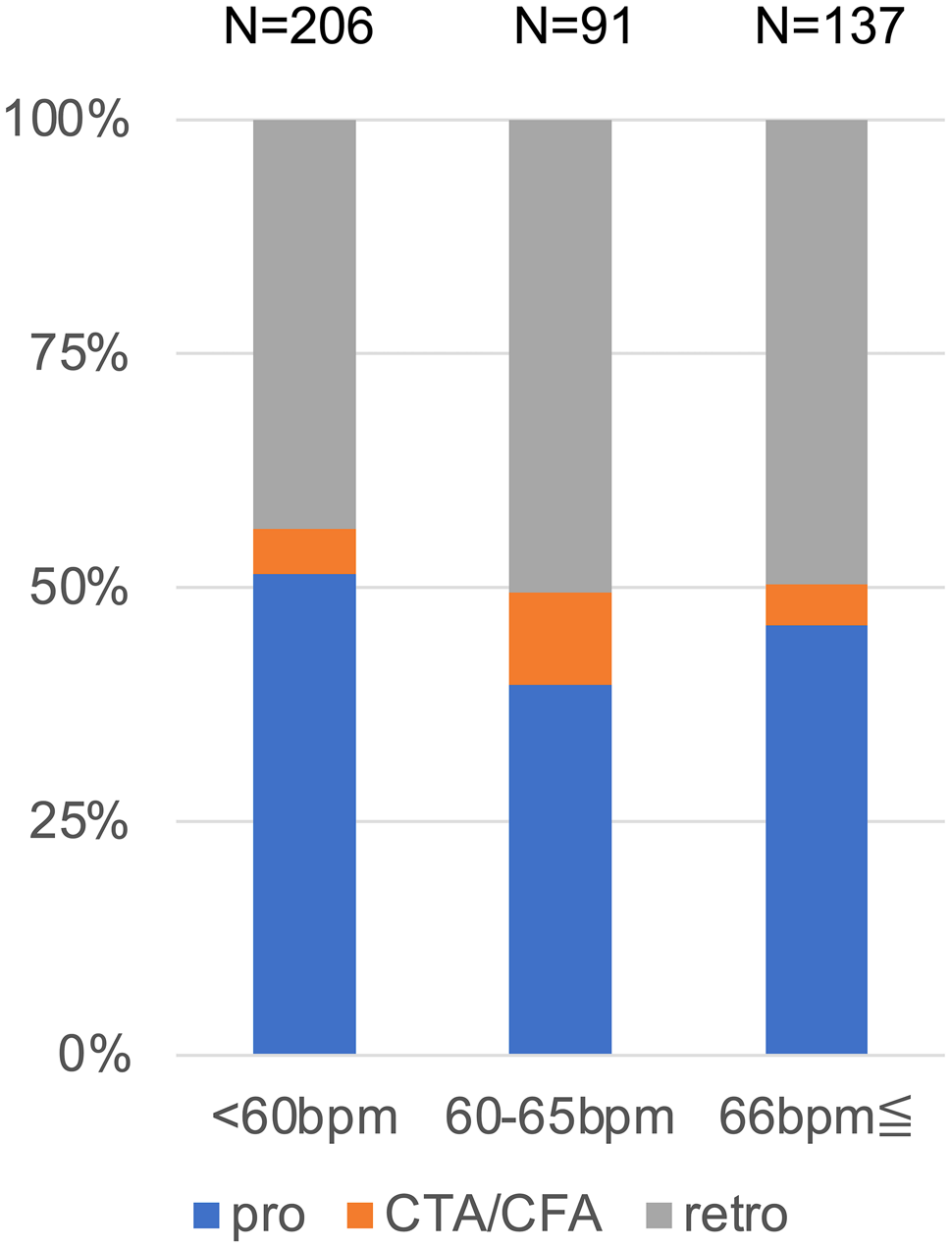
Scan techniques according to the heart rate pro, electrocardiogram-triggered prospective scanning; retro, electrocardiogram-gated retrospective scanning

The characteristics of the scan technique and tube potential at each hospital are depicted in Figs. 4 and 5, respectively, and are listed in ascending order using CTDIvol_CCTA_. ECG-triggered prospective scanning and low tube potentials were more frequently used in hospitals with lower CTDIvol_CCTA_. Figure 6 illustrates the scan techniques based on the heart rate in patients with normal sinus rhythm during the examination. ECG-gated retrospective scanning accounted for 44%, 51%, and 50% of patients with heart rate < 60 beats per minute (bpm), 61–65 bpm, and > 65 bpm, respectively.

### Predictors of CTDIvol in coronary CTA (CTDIvol_CCTA_)

To assess the factors that affect the radiation dose in coronary CTA, univariate and multivariate linear regression analyses were performed with an endpoint of 24 mGy, which was the third quartile of CTDIvol_CCTA_ in PROTECTION VI (Table 3). In univariate analysis, five of the eight variables were identified as predictors of radiation dose in coronary CTA and were subsequently used in the multivariable analysis. An increase in body weight by 10 kg and an increase in heart rate by 10 bpm were found to be associated with an increase in the radiation dose in the multivariable analysis, whereas a decrease in the tube potential by 10 kVp and the use of ECG-triggered prospective scanning were associated with a lower dose.

**Table 3:** Predictors of radiation dose from coronary computed tomography angiography

	Univariable analysis			Multivariable analysis		
Parameter	OR	95% CI	*p* value	OR	95% CI	*p* value
Patient weight (per 10 kg)	1.85	1.33–2.58	< 0.0001	8.6	2.46–30.01	0.0006
Patient height (per 10 cm)	1.01	0.79–1.30	0.908			
Heart rhythm	0.74	0.36–1.51	0.4088			
Sinus vs. others						
Heart rate (per 10 bpm)	1.5	1.19–1.87	0.0003	18.9	4.52–79.2	< 0.0001
Detector row	1.08	0.68–1.70	0.7476			
64 vs. more than 64						
Tube potential (per 10kVp)	1.55	1.36–1.78	< 0.0001	1.5	1.26–1.78	< 0.0001
ECG-controlled dose modulation (DM)	0.36	0.23–0.56	< 0.0001	0.92	0.52–1.61	0.773
ECG-triggered prospective scanning	0.07	0.04–0.12	< 0.0001	0.07	0.04–0.12	< 0.0001

Univariate and multivariate liner regression analyses were performed with an endpoint of 24 mGy, which represents the third quartile of CTDIvol_CCTA_ shown in PROTECTION VI

*CTA* computed tomography angiography, *ECG* electrocardiogram, *CTDIvol*_*CCTA*_ computed tomography dose index of coronary CTA

**Fig. 7:**
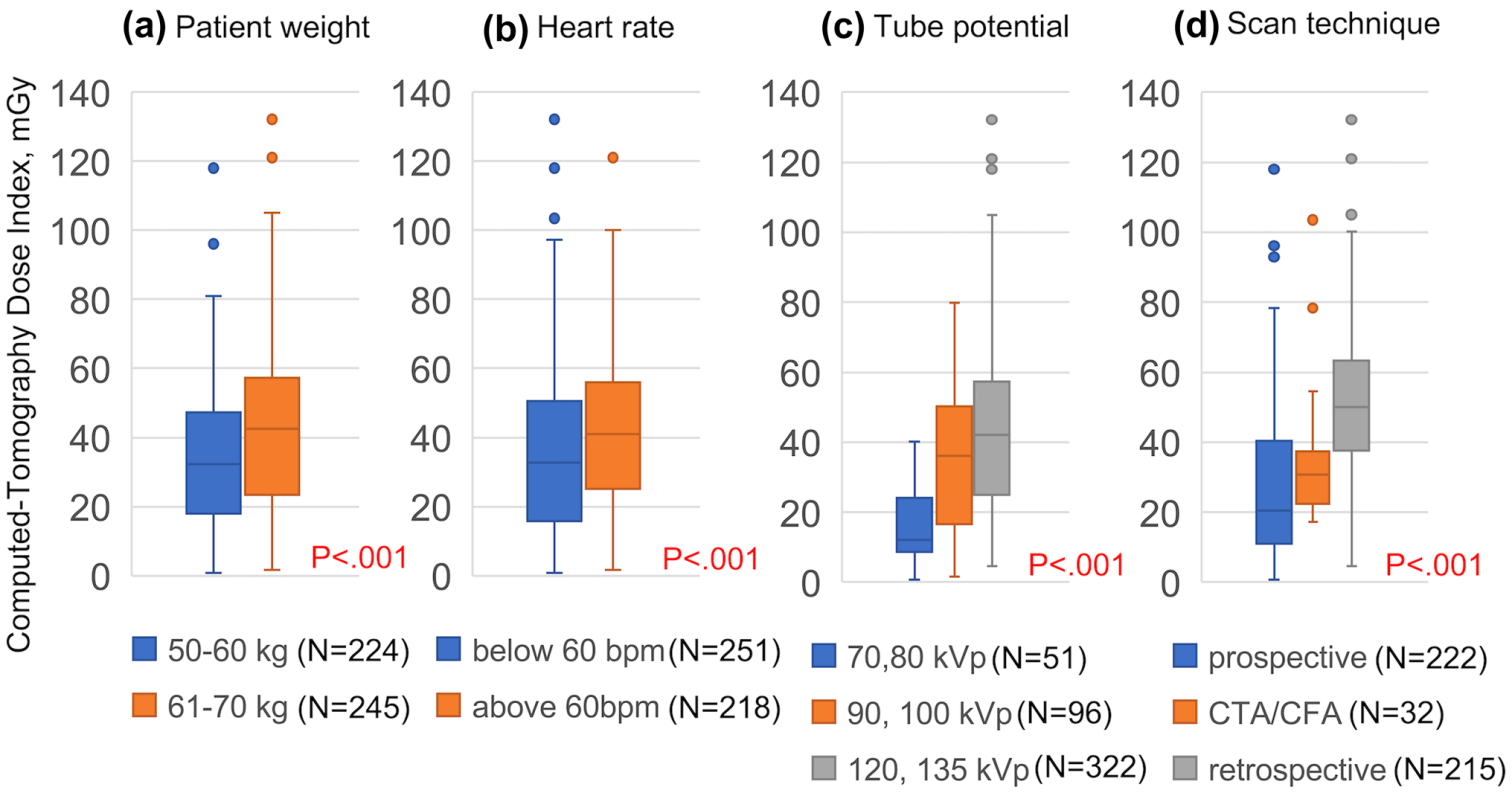
Differences in computed tomography dose index of coronary computed tomography angiography according to patient weight (**A**), heart rate (**B**), tube potential (**C**), and scan technique (**D**)

Figure 7 depicts the relationship between each variable and CTDIvol_CCTA_. For each variable, there were significant differences in the radiation dose between the groups; particularly, the tube potential and scan technique had an effect on the radiation dose during coronary CTA.

## Discussion

In this study, we performed a dose survey of cardiac CT scans across hospitals in Mie Prefecture, Japan. We noted the following observations. (1) The radiation doses of cardiac CT, both the total radiation dose of cardiac CT and the radiation dose of coronary CTA, were relatively well controlled compared to the DRL of the Japan DRLs 2020. However, the radiation dose was considerably higher than the DRL estimated in PROTECTION VI. (2) Low tube potentials and ECG-triggered prospective scanning are underutilized, and retrospective scans are often performed even in patients with adequately controlled heart rate. (3) Five of the 18 hospitals performed extensive imaging of the chest, abdomen, and pelvis in addition to coronary CTA during a single cardiac CT examination in > 50% of the cases.

Coronary CTA has a high diagnostic performance in detecting coronary artery stenosis and is widely used as a non-invasive imaging modality in daily clinical practice [1, 2]. All healthcare providers involved in performing CT have an obligation to optimize the radiation exposure using the ALARA principle while maintaining the diagnostic image quality [12]. Cardiac CT is no exception to this. In this survey, radiations from coronary CTA were relatively well controlled when compared to the Japan DRLs 2020. This might be due in part to the presence of a radiologist who had obtained a diploma from the Certification Board of Cardiovascular Computed Tomography (CBCCT), which certifies expertise in cardiovascular CT in the United States. In fact, the four hospitals that had established coronary CTA examination protocols under the radiologist's supervision were among the top six low-dose facilities in this dose survey. Currently, there is no certification system for coronary CTA in Japan, but such a certification system and educational programs for it would contribute to the nationwide dissemination of coronary CTA dose reduction techniques.

However, the median total DLP of cardiac CT was 786.1 mGy*cm (IQR: 560.6–1119.0 mGy*cm) and the median CTDIvol of coronary CTA was 32.8 mGy (IQR: 25.2–47.7 mGy), which are much higher than the median total DLP of 246 mGy*cm (IQR: 153–402 mGy*cm) and the median CTDIvol of coronary CTA of 14 mGy (IQR: 8–24 mGy) in PROTECTION VI [10]. This study demonstrated that increases in body weight and heart rate were independent predictors of a higher radiation dose during coronary CTA imaging. Conversely, low tube potential and ECG-triggered prospective scanning were independent predictors of lower radiation doses. The Society of Cardiovascular CT (SCCT) guidelines recommend low tube potential in patients with body weight < 100 kg or BMI < 30 kg/m^2^ [13]. Since only patients with 50–70 kg of body weight were included in this study, a low tube potential could potentially be applied to all coronary CTA scans. However, in reality, they are only utilized in approximately one-third of all cases. In the present survey, four centers used low tube potential in more than 90% of cases, probably because scanners at these institutions had an automatic tube voltage adjustment mechanism that suggested an appropriate tube voltage based on the patient's physique. On the other hand, many sites without such mechanism seem to continue using the manufacturer's default 120kVp protocol. In this regard, the initial settings proposed by the CT manufacturer’s application personnel at the time of CT installation may have a significant impact on the widespread use of low tube potential.

Regarding heart rate control for coronary CTA, the SCCT guidelines recommend that the target heart rate should be 60 bpm or less and beta-blockers can be considered to achieve short-term heart rate reduction. This is because in patients with a heart rate < 65 bpm and sinus rhythm, ECG-triggered prospective scanning can be used to reduce the dose while maintaining the image quality [14, 15]. Furthermore, Stocker et al. recently reported that coronary CTA image quality and radiation dose reduction were significantly better in patients with heart rate < 60 bpm than in patients with heart rate > 60 bpm even with modern high-temporal-resolution CT scanners [16]. It is important to emphasize that despite the SCCT recommendations for ECG-triggered prospective scanning, ECG-gated retrospective scanning was used in approximately half of the examinations in this study including patients with heart rate < 60 bpm (44%, 51%, and 50% of patients with heart rate < 60, 61–65, and > 65 bpm, respectively). The precise reason for the underutilization of ECG-triggered prospective scanning is unclear but may be related to the lack of data redundancy in ECG-triggered prospective scanning. ECG-gated retrospective scanning is the most reliable method for obtaining static coronary images that can reconstruct images of any cardiac phase and allows multi-segment reconstruction. For CT technologists, the choice of ECG-triggered prospective scanning may be associated with a sense of insecurity that the choice may lead to complaints about motion artifacts, stair-step artifacts, and a lack of systolic imaging and functional analysis. Therefore, the active involvement of radiologists in the development of cardiac CT protocols and the selection of optimal protocols for individual cases are extremely important for promoting radiation dose reduction.

The total DLP of cardiac CT in this study included the entire cardiac CT examination including those from positioning scans and non-contrast CT for calcium scoring. The present study revealed that extensive imaging, including contrast-enhanced CT for vascular evaluation from the chest to the pelvis, is often performed after coronary CTA. Given that the indications for such extensive scans were unclear (e.g., routine scan of the chest or abdomen) in some cases, each site should carefully evaluate whether such imaging is clinically indicated and avoid screening scans with inadequate risk assessments.

Furthermore, we would like to emphasize the importance of regional dose surveys. Previous dose surveys mainly collected data from large hospitals with radiologists. In the present survey, data were collected from all hospitals, regardless of whether they had a radiologist or not. We believe that providing feedback to local hospitals on the survey results, which better reflect the actual situation in the region, will provide a good opportunity to reconsider examination protocols for lower radiation exposure.

This study has several limitations. First, this was a retrospective study and not free from selection bias pertaining to local collaborators as we had requested them to select 30 consecutive patients during the period January 2021–December 2021. Second, the effect of dose reduction strategies on the diagnostic performance and image quality of coronary CTA was not evaluated. Third, width of padding is an important factor in the radiation dose of an ECG-triggered prospective scanning. However, because of the retrospective nature of this survey, it was difficult to collect information about padding. Forth, 10 of 44 facilities (23%) did not respond to the first survey in the present study for unknown reasons, which might indicate a low awareness of radiation dose exposure from CT examination including cardiac CT. Lastly, we did not investigate the institutions using CT systems with less than 64 detector rows. There are approximately 160 facilities in Mie prefecture that have CT systems with 4 or more slices, and it is possible that there are hospitals and clinics that perform coronary CTA with CT systems with less than 64 rows. However, the number of examinations at these facilities is likely to be extremely small, making them less significant for inclusion in the survey because all of the 22 facilities that were listed as capable of accepting ischemic heart disease in the prefecture’s medical plan had CT systems with 64 or more detector rows. Despite these limitations, this study clarified the predictors associated with radiation dose in cardiac CT in Japan by evaluating coronary CTA studies performed in Mie Prefecture in 2021. Although the results of this study are only the current situation in a single prefecture, it is highly likely that similar problems exist in other regions of Japan. This study will be a valuable reference for considering possible measures to reduce cardiac CT doses in Japan.

## Conclusion

The radiation dose of cardiac CT in Mie prefecture in 2021 was relatively well-controlled compared to that in the Japan DRLs 2020, but was substantially higher than the DRL in PROTECTION VI. The utilization of low tube potential and ECG-triggered prospective scanning following adequate heart rate control is essential for dose reduction in coronary CTA. The SCCT guidelines mention how and when to use low tube voltage, ECG-triggered prospective scanning and beta-blockers for heart rate control. Doctors and radiological technologists associated with the coronary CTA should be familiar with them and radiologists are especially expected to play a major role in optimizing cardiac CT protocols.

## Supplementary Information

Below is the link to the electronic supplementary material.

Supplementary file1 (PDF 32 KB)

Supplementary file2 (PDF 22 KB)
