# Supplementary material for: Radiation exposure in cardiac computed tomography imaging in Mie prefecture in 2021
Source: Jpn J Radiol. 2023 Jan 6;41(6):596–604. doi: 10.1007/s11604-022-01380-0 (PMC10232629; doi:10.1007/s11604-022-01380-0)
Supplement: Supplementary file 1 — Supplementary file1 (PDF 32 KB) [file 11604_2022_1380_MOESM1_ESM.pdf]

Supplementary Table. 1

Hospital	median $CTDIvol_{CCTA}$	Image interpreter	Major decision maker of scan methods	Supervision by Radiologist with CBCCT* diploma	Number of cardiac CT in 2021	Number of cardiac CT per month	Number of beds	Site experience in cardiac CT (years)	CT system
A	**3**	radiologist	radiologist	Yes	216	18	440	15	Dual-source
B	**13**	radiologist	radiologist	Yes	467	39	685	15	Dual-source
C	**19**	radiologist	radiological technologist		161	13	486	11	Area-detector
D	**20**	radiologist	radiological technologist		121	10	328	12	Area-detector
E	**25**	radiologist	radiological technologist	Yes	111	9	180	11	Standard
F	**27**	radiologist	radiological technologist	Yes	183	15	430	10	Area-detector
G	**30**	radiologist / cardiologist	radiological technologist		183	15	423	11	Area-detector
H	**32**	cardiologist	radiological technologist		282	24	379	14	Dual-source
I	**32**	radiologist / cardiologist	radiological technologist		721	60	568	12	Area-detector
J	**34**	radiologist	radiological technologist		122	10	335	12	Area-detector
K	**39**	cardiologist	radiological technologist		194	16	460	11	Standard
L	**48**	cardiologist	radiological technologist		273	23	647	15	Standard
M	**48**	radiologist	radiological technologist		180	15	200	8	Standard
N	**48**	radiologist	radiological technologist		363	30	400	15	Dual-source
O	**48**	cardiologist	radiological technologist		74	6	226	13	Area-detector
P	**55**	cardiologist	radiological technologist		490	41	45	17	Standard
Q	**68**	radiologist	radiological technologist		49	4	281	13	Standard
R	**71**	cardiologist	radiological technologist		312	26	199	14	Area-detector

* CBCCT, Certification Board of Cardiovascular Computed Tomography
