# Supplementary material for: Radiation exposure in cardiac computed tomography imaging in Mie prefecture in 2021
Source: Jpn J Radiol. 2023 Jan 6;41(6):596–604. doi: 10.1007/s11604-022-01380-0 (PMC10232629; doi:10.1007/s11604-022-01380-0)
Supplement: Supplementary file 2 — Supplementary file2 (PDF 22 KB) [file 11604_2022_1380_MOESM2_ESM.pdf]

Supplementary Table. 2

Vendor	CT scanner	n	number of detector row	minimum gantry rotation time (s)
Canon	Aquilion Prime	1	80	0.35
	Aquilion Prime SP	4	80	0.35
	Aquilion One *	3	320	0.35
	Aquilion One ViSION Edition*	2	320	0.275
	Aquilion One NATURE Edition*	2	320	0.275
Siemens	SOMATOM Definition Flash †	2	64	0.28
	SOMATOM Force †	1	96	0.25
	SOMATOM Drive †	1	64	0.28
	X.cite	1	64	0.3
Philips	Brilliance 64	1	64	0.42
	Brilliance iCT *	1	128	0.27
GE	Revolution Maxima	1	64	0.35

* Area (16 or 8 cm) detector CT

† dual-source CT
